# Acute Heart Failure developed as worsening of Chronic Heart Failure is associated with increased mortality compared to de novo cases

**DOI:** 10.1038/s41598-018-28027-3

**Published:** 2018-06-25

**Authors:** Vesna Degoricija, Matias Trbušić, Ines Potočnjak, Bojana Radulović, Sanda Dokoza Terešak, Gudrun Pregartner, Andrea Berghold, Beate Tiran, Saša Frank

**Affiliations:** 1 University of Zagreb School of Medicine, Šalata 3, 10000 Zagreb, Croatia; 2 University Hospital Centre Sisters of Charity, Department of Medicine, Vinogradska cesta 29, 10000 Zagreb, Croatia; 3 University Hospital Centre Zagreb, Kišpatićeva 12, 10000 Zagreb, Croatia; 4 Institute for Medical Informatics, Statistics und Documentation, Medical University of Graz, Auenbruggerplatz 2, 8036 Graz, Austria; 5 Clinical Institute for Medical and Chemical Laboratory Diagnostics, Medical University of Graz, Auenbruggerplatz 15, 8036 Graz, Austria; 6 Gottfried Schatz Research Center for Cell Signaling, Metabolism and Aging, Molecular Biology and Biochemistry, Medical University of Graz, Neue Stiftingtalstraße 6/6, 8010 Graz, Austria; 7 BioTechMed-Graz, Mozartgasse 12/II, 8010 Graz, Austria

**Keywords:** Outcomes research, Heart failure

## Abstract

Acute heart failure (AHF) emerges either *de novo* or from worsening of chronic heart failure (CHF). The aim of the present study was to evaluate the association between worsening of CHF and mortality in AHF patients. Out of 152 included AHF patients, 47 (30.9%) were *de novo* AHF patients and 105 (69%) were AHF patients with worsening of CHF. The proportion dying in hospital (19.0% vs. 4.3%, p = 0.023) and within 3 months after hospitalization (36.6% vs. 6.7%, p < 0.001) was significantly higher in AHF patients with worsening of CHF. Logistic regression analyses also showed a significant positive association of AHF emerging as worsening of CHF with hospital mortality [odds ratio (OR) and 95% confidence interval (CI): 5.29 (1.46–34.10), p = 0.029] and 3-month mortality [8.09 (2.70–35.03), p = 0.001]. While the association with hospital mortality was no longer significant after adjusting for comorbidities and clinical as well as laboratory parameters known to be associated with mortality in heart failure patients, the association with 3-month mortality remained significant. We conclude that compared to *de novo* AHF, AHF evolved from worsening of CHF is a more severe condition and is associated with increased mortality.

## Introduction

Heart failure (HF) is a frequent cause of death and disability worldwide^1^. According to the European Society of Cardiology (ESC) HF denotes an abnormality of the cardiac structure and function, resulting in failure of the heart to deliver oxygen at a rate commensurate with the requirements of the metabolizing tissues^2,3^. Acute heart failure (AHF) denotes the rapid onset, or a massive exaggeration of existing symptoms and signs of HF^3^. Accordingly, there are patients with de novo AHF and those with AHF as a consequence of the worsening of Chronic Heart Failure (CHF).

HF is a complex syndrome with a versatile underlying pathophysiology, highlighted by disturbed hemodynamics and a deranged metabolism^4^. Insufficient tissue perfusion due to impaired performance of the failing heart as well as tissue congestion due to venous volume overload, a consequence of right-sided HF, cause impaired intestinal nutrient absorption, diminished biosynthetic capacity of the liver^5^, and worsened renal function in HF^6,7^. Consequently, the decreased serum levels of cholesterol, increased concentrations of urea and creatinine, as well as wasting and chronic inflammation reflect the poor overall status associated with an increased mortality rate in HF patients^8^.

Considering the persistent hemodynamic and metabolic burden imposed on CHF patients we hypothesized that the clinical and laboratory characteristics are worse and the mortality rate higher in AHF patients with worsening of CHF compared to *de novo* AHF patients. Therefore, we compared the clinical and laboratory characteristics of patients with worsening of CHF with those of *de novo* AHF patients and evaluated the association between worsening of CHF and mortality in AHF patients.

## Results

### Clinical characteristics

The patients’ baseline characteristics have been described elsewhere^9^ and are shown in Table 1. Out of 152 included patients, 105 (69.1%) were patients with worsening of CHF and 47 (30.9%) were *de novo* AHF patients. As seen in Table 1, both groups were similar regarding age, sex, smoking status, body mass index (BMI) and New York Heart Association (NYHA) functional classification. Compared to *de novo* AHF patients, patients with worsening of CHF had a significantly lower mean arterial pressure (MAP) and heart rate (Table 1), whereas respiratory rate and the incidence of dyspnoea and ortopnoea were similar in both groups (Supplementary Table 1). Venous congestion, a consequence of right-sided HF, was more frequent in patients with worsening of CHF; this was exemplified by a significantly higher incidence of jugular venous distension (JVD), enlarged liver, and peripheral edema (Table 1). The incidence of ascites was higher, albeit not statistically significantly in patients with worsening of CHF compared to *de novo* AHF patients (Table 1). Furthermore, systolic pulmonary artery pressure (SPAP) was significantly higher in patients with worsening of CHF compared to *de novo* AHF patients, whereas ejection fraction (EF) (Table 1), the incidence of aortic insufficiency, aortic stenosis, and dilatation of right or left ventricle did not differ significantly between the groups (Supplementary Table 1).

**Table 1:** Baseline characteristics, vital signs and symptoms of AHF patients with worsening of CHF vs. *de novo* AHF patients.

	All AHF patients (N = 152)	De novo AHF (N = 47)	Worsening of CHF (N = 105)	p-value
Age (years)	77.3 (45.5–96.7)	75.7 (45.5–93.0)	77.5 (50.6–96.7)	0.312
Female	79 (52.0%)	20 (42.6%)	59 (56.2%)	0.160
BMI (kg/m^2^)	28.5 (16.3–43.5)	30.1 (16.3–38.6)	27.7 (17.1–43.5)	0.113
Weight (kg)	80.0 (40.0–144.0)	89.0 (50.0–125.0)	79.0 (40.0–144.0)	0.042
Smoking	38 (25.0%)	15 (31.9%)	23 (21.9%)	0.225
NYHA class				0.124
2	11 (7.2%)	6 (12.8%)	5 (4.8%)	
3	83 (54.6%)	27 (57.4%)	56 (53.3%)	
4	58 (38.2%)	14 (29.8%)	44 (41.9%)	
MAP (mmHg)	103.3 (53.3–160.0)	110.0 (70.0–160.0)	100.0 (53.3–156.7)	0.007
Heart rate (beats/min)	100.0 (36.0–160.0)	110.0 (36.0–160.0)	99.0 (50.0–150.0)	0.041
JVD	52 (34.2%)	10 (21.3%)	42 (40.0%)	0.027
Enlarged liver	53 (34.9%)	9 (19.1%)	44 (41.9%)	0.009
Peripheral edema	105 (69.1%)	26 (55.3%)	79 (75.2%)	0.022
Ascites	21 (13.8%)	4 (8.5%)	17 (16.2%)	0.309
EF (%)	45.0 (20.0–70.0)	45.0 (20.0–65.0)	41.0 (20.0–70.0)	0.677
SPAP (mmHg)	45.0 (35.0–80.0)	40.0 (35.0–60.0)	47.5 (35.0–80.0)	0.002

Data are presented as n (%) or as median and range (minimum to maximum). Differences between AHF patients with worsening of CHF and *de novo* AHF patients were tested with Fisher’s exact test or the Mann-Whitney U test, respectively.

AHF, acute heart failure; BMI, body mass index; CHF, chronic heart failure; EF, ejection fraction; JVD, jugular venous distension; MAP, mean arterial pressure; NYHA, New York Heart Association functional classification; SPAP, systolic pulmonary artery pressure.

Regarding comorbidities, the incidence of chronic obstructive pulmonary disease (COPD), chronic kidney disease (CKD), and cardiomyopathy (CM) were significantly higher, and the incidence of acute coronary syndrome (ACS) was significantly lower in AHF patients with worsening of CHF compared to *de novo* AHF patients (Supplementary Table 2). In contrast, the incidence of hyperlipidemia, hypercholesterolemia, hypertension, type 2 diabetes mellitus (T2DM) as well as coronary and peripheral atherosclerosis was similar in both groups.

### Laboratory parameters

The markers of kidney function urea and creatinine, were significantly higher and glomerular filtration rate (GFR) significantly lower in patients with worsening of CHF compared to *de novo* AHF patients (Table 2). While the N-terminal pro-Brain Natriuretic Peptide (NT-proBNP) and interleukin-6 (IL-6) levels were significantly higher, the levels of alanine aminotransferase (ALT), total cholesterol, low-density lipoprotein (LDL) cholesterol, and high-density lipoprotein (HDL) cholesterol, as well as leukocytes, and platelets were significantly lower in patients with worsening of CHF compared to *de novo* AHF patients (Table 2). Levels of aspartate transaminase (AST), C-reactive protein (CRP), triglycerides and erythrocytes did not differ significantly between groups (Table 2). While the levels of total serum protein and albumin, as well as glucose, sodium, and potassium were not significantly different between groups, the chloride levels were significantly lower in patients with worsening of CHF compared to *de novo* AHF patients (Supplementary Table 3).

**Table 2:** Laboratory parameters of AHF patients with worsening of CHF vs. *de novo* AHF patients.

	All AHF patients (N = 152)	De novo AHF (N = 47)	Worsening of CHF (N = 105)	p-value
GFR (ml/min/1.73 m^2^)	50.9 (15–0–105.7)	58.8 (20.0–105.7)	45.3 (15.0–104.8)	0.001
Urea (mmol/L)	8.0 (3.0–64.0)	7.0 (3.0–64.0)	9.0 (3.0–41.0)	0.003
Creatinine (µmol/L)	106.0 (53.0–273.0)	100.0 (53.0–255.0)	117.5 (59.0–273.0)	0.006
NT-proBNP (ng/mL)	9570 (171–70000)	4465 (171–70000)	12434 (291–70000)	<0.001
ALT (U/L)	23.0 (6.0–623.0)	25.0 (6.0–623.0)	21.0 (7.0–556.0)	0.034
AST (U/L)	27.0 (10.0–666.0)	28.0 (11.0–666.0)	27.0 (10.0–487.0)	0.536
IL-6 (pg/mL)	19.8 (0.4–300.0)	15.6 (0.4–300.0)	24.3 (1.2–300.0)	0.005
CRP (µg/mL)	9.4 (0.2–247.4)	5.7 (0.2–247.4)	10.6 (0.4–169.0)	0.189
Total cholesterol (mmol/L)	3.8 (1.7–9.1)	4.4 (2.5–9.1)	3.8 (1.7–7.7)	0.004
LDL cholesterol (mmol/L)	2.3 (0.8–6.3)	2.6 (1.3–6.3)	2.1 (0.8–6.0)	0.004
HDL cholesterol (mmol/L)	1.0 (0.3–3.6)	1.0 (0.4–1.9)	0.9 (0.3–3.6)	0.039
Triglycerides (mmol/L)	1.1 (0.5–4.3)	1.1 (0.6–4.3)	1.0 (0.5–3.2)	0.390
Erythrocytes (x 10^9^/L)	4.6 (2.5–6.6)	4.6 (3.6–5.9)	4.6 (2.5–6.6)	0.938
Leukocytes (x 10^9^/L)	9.7 (3.1–48.9)	11.2 (4.2–21.5)	9.4 (3.1–48.9)	0.017
Platelets (x 10^12^/L)	216.5 (61.0–999.0)	247.0 (71.0–999.0)	202.0 (61.0–444.0)	0.002

Data are presented as median and range (minimum to maximum). Differences between AHF patients with worsening of CHF and *de novo* AHF patients were tested with the Mann-Whitney U test.

ALT, alanine aminotransferase; AST, aspartate aminotransferase; AHF, acute heart failure; CHF, chronic heart failure; CRP, C-reactive protein; GFR, glomerular filtration rate; IL-6, interleukin-6; LDL, low-density lipoprotein; HDL, high-density lipoprotein; NT-proBNP, N-terminal pro brain natriuretic peptide.

### Mortality

Significantly more patients with AHF caused by worsening of CHF died in the hospital as well as within 3 months after hospitalization compared to *de novo* AHF patients (Table 3). In line with this, the univariable logistic regression analyses showed a significant positive association of worsening of CHF with hospital and 3-month mortality (Table 4). While this association did not remain significant for in-hospital mortality after adjusting for age, sex, BMI, NT-proBNP, MAP, GFR, and either urea, IL-6, and LDL cholesterol, or COPD, CKD, CM, ACS, and NYHA, the adjustment did not qualitatively change the results for 3-month mortality (Table 4).

**Table 3:** Mortality rate of AHF patients with worsening of CHF vs. *de novo* AHF patients.

	All AHF patients (N = 152)	De novo AHF (N = 47)	Worsening of CHF (N = 105)	p-value
Hospital mortality	22 (14.5%)	2 (4.3%)	20 (19.0%)	0.023
3-month mortality*	40 (27.4%)	3 (6.7%)	37 (36.6%)	<0.001

^*^N = 146 observations (45 de novo AHF, 101 worsening of CHF) available.

Data are presented as n (%). Differences between AHF patients with worsening of CHF and *de novo* AHF patients were tested with Fisher’s exact test; significant differences are depicted in bold. AHF, acute heart failure; CHF, chronic heart failure.

**Table 4:** Logistic regression analyses to assess the influence of the AHF class (worsening of CHF compared to *de novo* AHF) on hospital and 3-month mortality.

	OR (95% CI)	p-value	Events/N
**Hospital mortality**			
unadjusted	5.29 (1.46–34.10)	0.029	22/152
adjusted*	1.59 (1.33–11.64)	0.593	20/140
adjusted**	3.38 (0.65–27.86)	0.188	20/140
**3-month mortality**			
unadjusted	8.09 (2.70–35.03)	0.001	40/146
adjusted*	4.35 (1.20–21.72)	0.040	37/134
adjusted**	5.90 (1.53–31.68)	0.019	37/134

^*^Model was adjusted for age, sex, BMI, NT-proBNP, MAP, GFR, urea, IL-6, and LDL cholesterol.

**Model was adjusted for age, sex, BMI, NT-proBNP, MAP, GFR, COPD, CKD, CM, ACS, NYHA.

Data presented are ORs and 95% CIs for AHF as worsening of CHF vs. *de novo* AHF (reference) as well as the number of events and observations left in the analysis.

ACS, acute coronary syndrome; AHF, acute heart failure; BMI, body mass index; CHF, chronic heart failure; CI, confidence interval; CKD, chronic kidney disease; CM, cardiomyopathy; COPD, chronic obstructive pulmonary disease; GFR, glomerular filtration rate; IL-6, interleukin-6; LDL, low-density lipoprotein; MAP, mean arterial pressure; NT-proBNP, N-terminal brain natriuretic peptide; OR, odds ratio.

## Discussion

The present study clearly shows that AHF developed from CHF represents a more severe condition that is associated with higher mortality when compared to *de novo* AHF.

Compared to *de novo* AHF patients, AHF patients with worsening of CHF presented with markedly higher concentrations of NT-proBNP, a marker of heart congestion and elevated left-sided filling pressure^10^, indicating a more pronounced left ventricular dysfunction, accompanied by a lower MAP and higher SPAP and in turn, more severe right-sided HF with consequently more severe venous congestion.

Interestingly, despite a higher incidence of JVD, enlarged liver and peripheral edema, the body weight in AHF patients with worsening of CHF was significantly lower than in *de novo* AHF patients. This finding most probably reflects a more pronounced impairment of the intestinal absorption of nutrients due to a more severe intestinal edema, as well as a more severe chronic inflammation, the postulated common link between CHF, wasting syndrome and hypoalbuminemia^8,11,12^. In line with this, our AHF patients with worsening of CHF had lower serum levels of albumin (albeit not statistically significant) and significantly higher concentrations of IL-6 when compared to *de novo* AHF patients.

It is generally accepted that the augmented translocation of bacterial endotoxins from the edematous intestine into the circulation^13^ as well as insufficient binding and neutralization of endotoxines by circulating lipoproteins, whose levels are decreased in HF^14,15^, underlie the persistent inflammatory response in HF^16^. Additionally, the pro-inflammatory activation of venous endothelial cells by circumferential stretch due to congestion contributes to the chronic inflammation in HF^17^. Accordingly, lower serum levels of LDL cholesterol and HDL cholesterol together with a more severe congestion, in patients with worsening of CHF compared to *de novo* AHF patients may well explain the more pronounced inflammatory response, reflected by higher IL-6 levels.

The augmented inflammatory response, which is known to promote the formation and clearance of leukocyte - platelet complexes^18,19^, is most likely responsible for the lower leukocyte and platelet levels in our AHF patients with worsening of CHF.

It is well established that decreased cholesterol and lipoprotein serum levels are strongly and independently associated with increased mortality in HF^8,14^. In contrast to total, LDL, and HDL cholesterol, which were significantly lower in patients with worsening of CHF, triglyceride levels were similar in both studied groups.

Serum levels of lipids and lipoproteins are determined by their intestinal absorption, hepatic synthesis, and removal from the circulation by various catabolic routes. Accordingly, modulation of these physiological processes by the underlying AHF pathophysiology, such as congestion, which was more severe in AHF patients with worsening of CHF, may explain the different levels of total, LDL, and HDL cholesterol in the studied groups. Similar triglyceride levels in the two studied groups may indicate either that the triglyceride synthesis is not affected by the underlying AHF pathophysiology or that the decreased triglyceride synthesis is counterbalanced by a likewise decreased triglyceride catabolism. The latter possibility is likely, because lipoprotein lipase, a serum enzyme responsible for triglyceride degradation, is decreased by inflammation^20^, which indeed was more severe in AHF patients with worsening of CHF than in *de novo* AHF patients.

Renal dysfunction, also a strong predictor of mortality in HF^21,22^, was more pronounced in our AHF patients with worsening of CHF than in *de novo* AHF patients. This was reflected by a markedly decreased GFR and concomitantly increased serum levels of urea and creatinine. The reduction in GFR may be due to several factors including kidney hypoperfusion^23^, renal co-morbidities^24^, renal venous congestion^25,26^, or inflammatory cytokines such as IL-6^27^. Accordingly, a higher incidence of CKD, together with a more severe venous congestion and a more pronounced inflammatory response, as well as higher serum levels of NT-proBNP, an established predictor of worsening renal function in CHF^28^, may well explain the more severe impairment of renal function in our AHF patients with worsening of CHF compared to *de novo* AHF patients.

Besides their deleterious effects on the kidneys, inflammatory cytokines such as IL-6 exert a direct detrimental effect on the myocardial structure and function by promoting oxidative stress and endothelial dysfunction, thus contributing to the progression of HF^29^. Therefore, a more pronounced inflammatory state, reflected by higher IL-6 levels, seems to be an important underlying pathophysiology responsible for worse clinical status of our AHF patients with worsening of CHF compared to *de novo* AHF patients.

The above-mentioned differences between the groups regarding the clinical characteristics observed in our AHF patients with worsening of CHF translated into a higher mortality rate, whereby the impact on 3-month mortality was more pronounced than the impact on hospital mortality. This is in line with two recent reports showing a strong association of the chronicity of HF with 6-month and 3-year mortality, but only a weak association with 1-month mortality^30,31^. In the present study, the strong association of worsening of CHF with 3-month mortality was weakened, but remained significant upon adjustment for comorbidities and established risk factors in HF^8,32,33^. This implies that other features inherent to this group of patients underlie their increased mortality compared to de novo AHF patients.

There are several limitations to our present study: The design precludes conclusions on the pathophysiological mechanisms in terms of cause and consequence, or temporal development and onset of signs, and symptoms associated with worsened status and increased mortality. Furthermore, our data do not facilitate the examination whether the duration of HF chronicity affects mortality rates. Finally, the limited number of participants in this monocentric study influences the statistical power of our analyses. Therefore, further large studies are needed to confirm our results.

Based on our results, we conclude that AHF developed from CHF is a more severe condition than *de novo* AHF and is associated with higher mortality rates.

## Methods

### Study design and patients

Study design, inclusion and exclusion criteria as well as patient characteristics for our AHF cohort have been reported previously^9,34^. In short, we performed a prospective observational study on hospitalized patients with AHF in Zagreb, Croatia, between 2013 and 2015. Written informed consent was obtained from each patient and the study, which was approved by the Ethics Committees of the University Hospital Centre Sisters of Charity, Zagreb, Croatia and the Medical University of Graz, Austria, was conducted in adherence to the ethical guidelines of the Declaration of Helsinki^35^. All patients were treated according to the ESC Guidelines for AHF^3,36^.

### Measurements of SPAP

SPAP was approximated the tricuspid valve velocity, estimated central vein pressure (resembling right atrium pressure), and Bernoulli equation from a Doppler echocardiography.

### Laboratory procedures

The collection of blood samples, standard laboratory methods, and the determination of the NT-proBNP concentration have been described in our previous reports on our AHF cohort^34,37^. The levels of alanine aminotransferase (ALT) and aspartate aminotransferase (AST) were determined with the Abbott Architect c8000 (Chicago, IL, USA).

### Statistical analyses

Categorical data are shown as absolute and relative frequencies, whereas continuous data are summarized as median and range (minimum to maximum) due to the skewed distribution of many of the laboratory parameters. Patients with *de novo* AHF and with worsening of CHF were compared by Mann-Whitney U test or Fisher’s exact test. The impact of worsening of CHF as compared to *de novo* AHF on hospital and 3-month mortality was assessed by logistic regression analysis. In addition to a univariable model, we also adjusted for age, sex, BMI, NT-proBNP, GFR, MAP, urea, IL-6, and LDL cholesterol, as well as for age, sex, BMI, NT-proBNP, MAP, GFR, COPD, CKD, CM, ACS, and NYHA, the comorbidities as well as clinical and laboratory parameters known to be associated with mortality in HF patients. We checked the variance inflation factor to prevent multi-collinearity among the covariates. Results are presented as odds ratios (OR) with the respective 95% confidence interval (CI). All analyses are exploratory and a p-value of <0.05 was considered significant. R version 3.4.2. was used for all statistical analyses.

## Electronic supplementary material

Supplementary Tables

## Data Availability

All data generated or analysed during this study are included in this manuscript and accompanied Supplementary materia.
