# Author Correction: Acute Heart Failure developed as worsening of Chronic Heart Failure is associated with increased mortality compared to de novo cases

**DOI:** 10.1038/s41598-019-44069-7

**Published:** 2019-06-05

**Authors:** Vesna Degoricija, Matias Trbušić, Ines Potočnjak, Bojana Radulović, Sanda Dokoza Terešak, Gudrun Pregartner, Andrea Berghold, Beate Tiran, Saša Frank

**Affiliations:** 1 University of Zagreb School of Medicine, Šalata 3, 10000 Zagreb, Croatia; 2 University Hospital Centre Sisters of Charity, Department of Medicine, Vinogradska cesta 29, 10000 Zagreb, Croatia; 3 University Hospital Centre Zagreb, Kišpatićeva 12, 10000 Zagreb, Croatia; 4 Institute for Medical Informatics, Statistics und Documentation, Medical University of Graz, Auenbruggerplatz 2, 8036 Graz, Austria; 5 Clinical Institute for Medical and Chemical Laboratory Diagnostics, Medical University of Graz, Auenbruggerplatz 15, 8036 Graz, Austria; 6 Gottfried Schatz Research Center for Cell Signaling, Metabolism and Aging, Molecular Biology and Biochemistry, Medical University of Graz, Neue Stiftingtalstraße 6/6, 8010 Graz, Austria; 7 BioTechMed-Graz, Mozartgasse 12/II, 8010 Graz, Austria

Correction to: *Scientific Reports* 10.1038/s41598-018-28027-3, published online 25 June 2018

In the original version of this Article, Ines Potočnjak and Sanda Dokoza Terešak were incorrectly affiliated with ‘University of Zagreb School of Medicine, Šalata 3, 10000 Zagreb, Croatia’. The correct affiliation is listed below.

University Hospital Centre Sisters of Charity, Department of Medicine, Vinogradska cesta 29, 10000 Zagreb, Croatia

This error has now been corrected in the PDF and HTML versions of the Article.

