# Supplementary material for: Acute Heart Failure developed as worsening of Chronic Heart Failure is associated with increased mortality compared to de novo cases
Source: Sci Rep. 2018 Jun 25;8:9587. doi: 10.1038/s41598-018-28027-3 (PMC6018547; doi:10.1038/s41598-018-28027-3)
Supplement: Supplementary file 1 — Supplementary Tables [file 41598_2018_28027_MOESM1_ESM.pdf]

Acute Heart Failure developed as worsening of Chronic Heart Failure is associated with increased mortality compared to de novo cases

Vesna Degoricija, Matias Trbušić, Ines Potočnjak, Bojana Radulović, Sanda Dokoza Terešak, Gudrun Pregartner, Andrea Berghold, Beate Tiran and Saša Frank

Supplementary Table 1. Vital signs and symptoms of AHF patients with worsening of CHF vs. *de novo* AHF patients

	All AHF patients (N=152)	De novo AHF (N=47)	Worsening of CHF (N=105)	p-value
**Respiratory rate (breaths/min.)**	28.0 (12.0-75.0)	28.0 (12.0-75.0)	28.0 (15.0-50.0)	0.561
**Dispnoea**	143 (94.1%)	44 (93.6%)	99 (94.3%)	1.000
**Ortopnoea**	132 (86.8%)	38 (80.9%)	94 (89.5%)	0.193
**Aortic insufficiency**	39 (27.7%)	10 (22.7%)	29 (29.9%)	0.422
**Aortic stenosis**	43 (31.6%)	11 (25.0%)	32 (34.8%)	0.325
**RV dilatation**	42 (29.2%)	9 (20.5%)	33 (33.0%)	0.164
**LV dilatation**	57 (39.6%)	17 (38.6%)	40 (40.0%)	1.000

Data are presented as n (%) or as median and range (minimum to maximum). Differences between AHF patients with worsening of CHF and *de novo* AHF patients were tested with Fisher's exact test or the Mann-Whitney U test, respectively.

AHF, acute heart failure; CHF, chronic heart failure; LV, left ventricle; RV, right ventricle;

Supplementary Table 2. Comorbidities of AHF patients with worsening of CHF vs. *de novo* AHF patients

	All AHF patients (N=152)	De novo AHF (N=47)	Worsening of CHF (N=105)	p-value
**Hyperlipidemia**	60 (39.5%)	20 (42.6%)	40 (38.1%)	0.720
**Hypercholesterolemia**	59 (38.8%)	20 (42.6%)	39 (37.1%)	0.590
**Hypertension**	136 (89.5%)	44 (93.6%)	92 (87.6%)	0.393
**T2DM**	78 (51.7%)	23 (48.9%)	55 (52.9%)	0.726
**COPD**	41 (27.0%)	6 (12.8%)	35 (33.3%)	**0.010**
**CKD**	50 (32.9%)	7 (14.9%)	43 (41.0%)	**0.001**
**CM**	113 (74.3%)	23 (48.9%)	90 (85.7%)	**<0.001**
**ACS**	24 (15.8%)	13 (27.7%)	11 (10.5%)	**0.014**
**Coronary atherosclerosis**	69 (45.4%)	16 (34.0%)	53 (50.5%)	0.078
**Peripheral atherosclerosis**	37 (32.2%)	10 (30.3%)	27 (32.9%)	0.829

Data are presented as n (%) or as median and range (minimum to maximum). Differences between AHF patients with worsening of CHF and *de novo* AHF patients were tested with Fisher's exact test or the Mann-Whitney U test, respectively; significant differences are depicted in bold.

ACS, acute coronary syndrome; AHF, acute heart failure; CHF, chronic heart failure; CKD, chronic kidney disease; CM, cardiomyopathy; COPD, chronic obstructive pulmonary disease; T2DM, Type 2 Diabetes Mellitus;

Supplementary Table 3. Serum glucose, proteins and electrolytes of AHF patients with worsening of CHF vs. *de novo* AHF patients

	All AHF patients (N=152)	De novo AHF (N=47)	Worsening of CHF (N=105)	p-value
**Glucose (mmol/L)**	8.2 (4.1-31.6)	8.4 (4.7-29.0)	7.9 (4.1-31.6)	0.328
**Serum protein (g/L)**	68.0 (31.0-87.0)	69.0 (41.0-85.0)	67.5 (31.0-87.0)	0.097
**Albumin (g/L)**	40.0 (21.0-72.0)	42.0 (24.0-62.0)	39.0 (21.0-72.0)	0.055
**Sodium (mmol/L)**	140.0 (115.0-148.0)	141.0 (125.0-146.0)	140.0 (115.0-148.0)	0.240
**Potassium (mmol/L)**	4.4 (3.1-6.3)	4.2 (3.2-5.6)	4.4 (3.1-6.3)	0.078
**Chloride (mmol/L)**	104.0 (77.0-114.0)	104.0 (84.0-114.0)	103.0 (77.0-112.0)	**0.033**

Data are presented as median and range (minimum to maximum). Differences between AHF patients with worsening of CHF and *de novo* AHF patients were tested with the Mann-Whitney U test; significant differences are depicted in bold.

AHF, acute heart failure; CHF, chronic heart failure;
